# Targeting HOX and PBX transcription factors in ovarian cancer

**DOI:** 10.1186/1471-2407-10-89

**Published:** 2010-03-10

**Authors:** Richard Morgan, Lynn Plowright, Kevin J Harrington, Agnieszka Michael, Hardev S Pandha

**Affiliations:** 1 Postgraduate Medical School, University of Surrey, Guildford, UK; 2 Targeted Therapy Team, Chester Beatty Laboratories, The Institute of Cancer Research, London, UK

## Abstract

**Backgound:**

Ovarian cancer still has a relatively poor prognosis due to the frequent occurrence of drug resistance, making the identification of new therapeutic targets an important goal. We have studied the role of *HOX *genes in the survival and proliferation of ovarian cancer cells. These are a family of homeodomain-containing transcription factors that determine cell and tissue identity in the early embryo, and have an anti-apoptotic role in a number of malignancies including lung and renal cancer.

**Methods:**

We used QPCR to determine HOX gene expression in normal ovary and in the ovarian cancer cell lines SK-OV3 and OV-90. We used a short peptide, HXR9, to disrupt the formation of HOX/PBX dimers and alter transcriptional regulation by HOX proteins.

**Results:**

In this study we show that the ovarian cancer derived line SK-OV3, but not OV-90, exhibits highly dysregulated expression of members of the *HOX *gene family. Disrupting the interaction between HOX proteins and their co-factor PBX induces apoptosis in SK-OV3 cells and retards tumour growth *in vivo*.

**Conclusion:**

HOX/PBX binding is a potential target in ovarian cancer

## Background

Ovarian cancer is a relatively uncommon malignancy, accounting for 4% of all cancers in the western world and 5% of all cancer deaths in women, but the mortality from this disease has improved little in the last 30 years [1]. Detection of ovarian cancer is often at a relatively advanced stage of the disease due to a lack of specific symptoms, and the overall survival for women diagnosed with stage III or IV ovarian cancer varies from 18.6-46.7%. Treatment is usually a combination of surgery and chemotherapy, frequently using carboplatin, but recurrence and resistance is commonly observed with an associated high mortality in these cases [1].

In a similar manner to other cancers, ovarian cancers are known to over express a number of genes involved in early development. These include the *HOX *genes, a family of homeodomain-containing transcription factors that define the identity of cells and tissues during early development [2]. There are 39 *HOX *genes in mammals, divided into four groups (A-D) in tightly linked clusters on different chromosomes [3]. Whilst some *HOX *genes have distinct functions in specific contexts, many others have overlapping or redundant functions during early development [4], in haematological malignancies [5], and in a number of other cancers including melanoma [6] and renal cancer [7], where the *HOX *genes have a potent anti-apoptotic function. This redundancy in HOX function is based in part upon the binding of similar DNA sequences, and also on the interaction of HOX proteins with a common set of co-factors including PBX. PBX modifies the DNA binding specificity of HOX proteins, influences the regulation of transcription, and is required for many aspects of HOX function [8].

Changes in *HOX *gene expression have also been observed in ovarian cancer [9-11]. Over expression of *HOXA9*, *HOXA10 *or *HOXA11 *results in a serous, endometrioid-like or mucinous-like phenotype respectively [9]. In addition, *HOX *genes have been shown to influence the ability of ovarian cancer cells to invade other tissues [11], and to promote tumour growth [10]. In this study we have addressed whether *HOX *genes have an anti-apoptotic effect in ovarian cancer. We show that *HOX *expression in the ovarian cancer cell line SK-OV3 is highly dysregulated compared to normal ovarian tissue. Furthermore, interfering with *HOX *function using the PBX-binding peptide HXR9 [6] triggers apoptosis in SK-OV3 cells both *in vitro *and *in vivo*.

## Methods

### Maintenance of SK-OV3 and OV-90 in culture

The human ovarian adenocarcinoma-derived cell lines SK-OV3 and OV-90 were obtained from the American Type Culture Collection (LGC Promochem, Teddington, UK). The cells were cultured in McCoy's 5A modified medium (Sigma, Poole, UK) supplemented with 10% (v/v) heat-inactivated foetal bovine serum (Invitrogen Ltd, Paisley, UK) and 1% penicillin (10,000 U/ml)/streptomycin (10 mg/ml) (Sigma). Cell cultures were maintained at 37°C in a humidified, 5% CO_2 _incubator.

### Semi quantitative PCR

Total RNA from normal human ovary tissue was purchased from Applied Biosystems (Warrington, UK). RNA was isolated from SK-OV3 and OV-90 cells using the RNeasy^® ^Plus Mini Kit (Qiagen Ltd, Crawley, UK) according to the manufacturer's instructions. cDNA was synthesised from RNA using the Cloned AMV First Strand Synthesis Kit (Invitrogen) following the manufacturer's protocol. Semi-quantitative RT-PCR was performed using the Stratagene MX3005P Real Time PCR machine (Agilent Technologies UK Ltd, Stockport, UK) and SYBR^® ^Green JumpStart™ Taq ReadyMix™ (Sigma). Oligonucleotide primers were designed to facilitate the unique amplification of *β-actin*, *c-Fos *and each *HOX *gene. Relative expression was calculated using the Livak comparative Ct method (2^-ΔΔCt^) [10].

### Analysis of cell death and apoptosis

Cell viability was measured via the MTS assay (Promega, Southampton, UK) according to the manufacturer's instructions. Briefly, cells were plated in a 96-well plate at a concentration of 1 × 10^5 ^cells/ml and incubated for 24 hours. Cells were treated overnight with the active peptide HXR9 or the control peptide CXR9 at a range of dilutions. The IC_50 _of the cells was determined by plotting a dose-response curve. To detect morphological changes consistent with apoptosis, cells were harvested by incubating in trypsin-EDTA (Sigma) at 37°C until detached and dissociated. Apoptotic cells were identified using a Beckman Coulter Epics XL flow cytometer (argon laser, excitation wavelength 488 nm, FL-2 and FL-4 detectors) and the Annexin V-PE apoptosis detection kit (BD Pharmingen).

### Imaging of the cells

For phase contrast micrographs, cells were plated into 60 mm tissue culture dishes at a concentration of 1 × 10^5 ^cells/ml and incubated at 37°C for 24 hours. Cells were treated overnight with HXR9 (120 μM) or CXR9 (120 μM). The cells were washed twice with PBS, visualised using a Nikon Eclipse TE100 inverted microscope and images recorded using a Nikon DS-L camera and capture software (Nikon Instruments Europe B.V., Amstelveen, The Netherlands).

For fluorescent images of HXR9 localisation, 35 mm culture dishes were seeded with 2 × 10^5 ^cells and incubated as previously described. The cultures were treated with HXR9-FITC conjugate (30 μM) for 1 hour. The cells were fixed with 10% formalin in neutral-buffered saline (Sigma). Cells were visualised using a Nikon Eclipse TE 2000-S fluorescent microscope and images recorded using a DMX 1200F camera and NIS Elements capture software (Nikon Instruments).

### Detection of PARP cleavage by western blotting

SK-OV3 cells were seeded at a concentration of 4 × 10^4 ^cells/ml on 60 mm tissue culture dishes and were incubated at 37°C. When cells approached 75% confluence, cultures were treated overnight with HXR9 (120 μM) or CXR9 (120 μM). Lysates were prepared with RIPA buffer containing protease and phosphatase inhibitor cocktails and EDTA (Perbio Science UK Ltd, Cramlington, UK). The protein concentration was quantified using the bicinchoninic acid assay (Perbio) according to the manufacturer's instructions. Proteins were resolved by 10% SDS-polyacrylamide gel electrophoresis and transferred to polyvinylidene fluoride membrane. Anti-PARP antibody (Biomol International, Exeter, UK) was used to detect both cleaved and full-length PARP. To visualise proteins, the ECL Western blotting detection system (Amersham Biosciences, Little Chalfont, UK) was used.

### Mice and *in vivo *trial

All animal experiments were conducted in accordance with the United Kingdom Co-ordinating Committee on Cancer Research (UKCCCR) guidelines for the Welfare of Animals in Experimental Neoplasia [12] and approved by the St. George's Hospital Medical School Ethical Review Committee. The mice were kept in positive pressure isolators in 12 hour light/dark cycles and food and water were available *ad libitum*.

Athymic nude mice were inoculated subcutaneously with a suspension of 2.5 × 10^6 ^SK-OV3 cells in culture media (100 μl). Once tumours reached volumes of approximately 100 mm^3^, mice received an initial dose of 100 mg/Kg CXR9 or HXR9 intravenously, with subsequent dosing of 10 mg/Kg twice weekly. Each treatment group contained 10 mice. The mice were monitored carefully for signs of distress, including behavioural changes and weight loss.

An additional 10 mice were included in each treatment group to allow the amount of HXR9 peptide in tumours to be assessed. Tumours were excised from three mice at 3, 12 and 24 hours after iv administration of 100 mg/Kg HXR9. Total cellular protein was extracted and 10 μg were dot blotted on nitrocellulose membrane and probed with a rabbit polyclonal anti-HXR9 antibody. Quantification was achieved using a standard series of HXR9 dilutions.

### Statistical analysis

Data are given as means ± SEM of at least three independent experiments. Significant effects were determined by 2-tailed Student's *t *tests (p < 0.05), or the Mann-Whitney test for mouse tumour modelling experiments. For the analysis of the QPCR for HOX gene expression the Bonferroni correction was applied to account for multiple comparisons. There are 39 HOX genes thus the limit for a significant result is p < 0.0013.

## Results

### *Hox *gene expression in normal ovarian tissue and ovarian cancer cell lines

**Figure 1:**
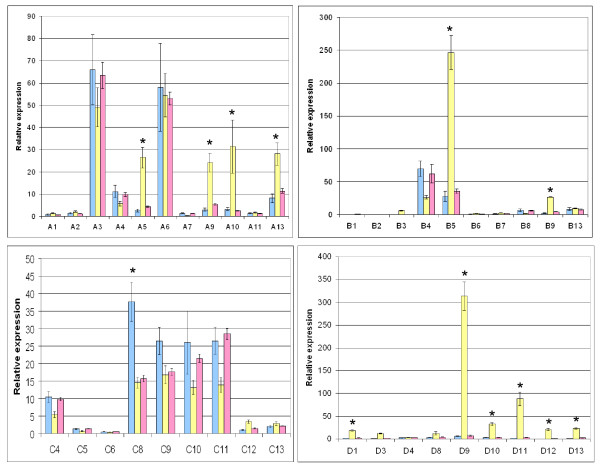
**HOX gene expression in normal ovary and in the ovarian cancer derived cell lines SK-OV3 and OV-90 cells**. Gene expression was determined by QPCR and expressed as a ratio to the amount of expression of the housekeeping gene, *β-actin*. Abbreviations are used for each *HOX *gene shown on the x-axis such that, for example, *HOXA1 *is represented by 'A1'. SEM is indicated by the error bars. P-values of less than 0.0013 (Bonferroni correction for multiple hypothesis testing) for expression in SK-OV3 cells compared to that in normal ovary are denoted *.

Previous studies have examined the expression of *HOX *genes in ovarian tumours but to date this had not been done in a comprehensive manner, looking at all 39 members of this family. Thus, we measured the relative expression of *HOX *genes in normal ovarian tissue and in the derived cell lines SK-OV3 and OV-90 using semi-quantitative PCR (Fig 1). This revealed that multiple *HOX *genes had a dysregulated pattern of expression in SK-OV3 cells, but generally not in OV-90 cells, in comparison to the normal ovary. In SK-OV3 the A family of the *HOX *genes showed a statistically significant up regulation of *HOXA5, HOXA9*, and *HOXA13*. The *HOXB *and *HOXD *families were mainly expressed only in SK-OV3 cells; *HOXB5*, *HOXD9*, and *HOXD11 *showed significantly increased expression.

#### Targeting HOX/PBX dimers in SK-OV3 and OV-90

**Figure 2:**
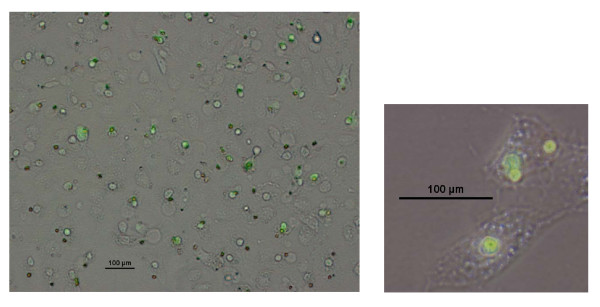
**Uptake of fluorescently labelled HXR9 by SK-OV3 cells**. Cells were incubated for one hour with an HXR9-FITC conjugate (30 μM) (green). HXR9 is confined to the nucleus in the majority of cells.

**Figure 3:**
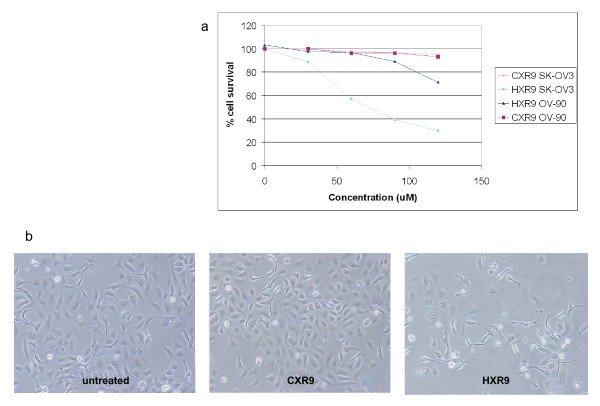
**(a) HXR9 toxicity was determined using the MTS assay**. SK-OV3 and OV-90 cells were treated overnight with 30, 60, 90 and 120 μM of either CXR9 or HXR9. A sample of the same cell suspension was used as a "cell-only" control. Results here are presented as an average of three independent experiments ± SEM. The IC_50 _for SK-OV3 was estimated from the graph to be 70 μM, no value could be determined for OV-90. **(b) ***Phase contrast micrographs of SKOV-3 cells treated overnight with 60 μM of HXR9 or CXR9*. In HXR9 treated cultures, fewer number of adherent cells were evident and many detached, dead cells were apparent. (×120 magnification)

Results from previous studies [9,11] together with our data show that many *HOX *genes are upregulated in ovarian cancer. We therefore decided to study the role of *HOX *genes in SK-OV3 and OV-90 cells by interfering with HOX function. Previous work has shown that disrupting the interaction between HOX proteins and their PBX co-factor can trigger apoptosis in melanoma and renal cancer cells [6,7]. PBX binding to HOX modifies the selection of DNA binding sites and hence the identity of *HOX *target genes. A short peptide, HXR9, was previously shown to disrupt the formation of HOX/PBX dimers, alter transcriptional regulation by HOX proteins and thereby induce apoptosis [6,7]. HXR9 contains nine repeated arginine residues to facilitate cellular uptake, probably through endocytosis [13]. In order to show that HXR9 was able to enter cells, SK-OV3 cultures were incubated with a fluorescently labelled version of the peptide. This indicated that HXR9 was indeed taken up by SK-OV3 cells and tended to accumulate in the nucleus (Fig 2). Furthermore, HXR9 killed SK-OV3 cells with an IC_50 _of approximately 70 μM, although it did not kill OV-90 cells (Fig 3a). Cell death in SK-OV3 cells was accompanied by characteristic changes in cellular morphology (Fig 3b).

#### HXR9 treatment causes apoptosis

**Figure 4:**
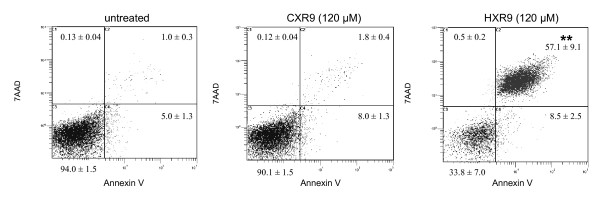
**Fluorescence activated cell sorter (FACS) analysis of Annexin V-PE stained SK-OV3 cells**. Induction of apoptosis in SK-OV3 cells treated overnight with HXR9 or CXR9 (120 μM) was compared with untreated cells. The results are presented as mean values ± SEM of five independent experiments. ** indicates a significant difference between treatment groups, p < 0.001.

Flow cytometric analysis was used to distinguish between apoptotic and necrotic cell death. SK-OV3 cells were stained with Annexin-V-PE to detect the externalisation of phosphatidylserine, which occurs during the early stages of apoptosis. Cells were counterstained with the vital dye 7AAD. When compared with controls, a significantly higher proportion of SK-OV3 cells treated with HXR9 were shown to be in late apoptosis (*P *< 0.001) but few cells were found to be in the early apoptotic or necrotic state (Fig 4).

**Figure 5:**
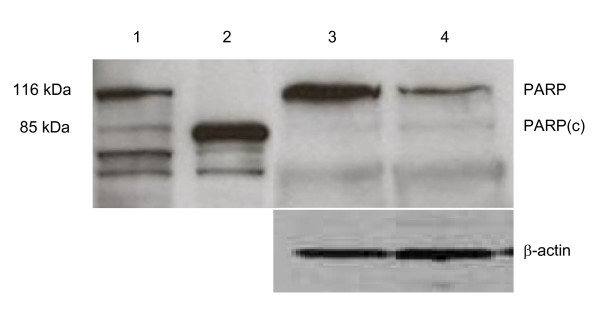
**PARP cleavage induced by HXR9**. Lysates of CXR9-treated and HXR9-treated SK-OV3 cells were probed with an anti-PARP antibody. A lysate of etoposide treated HL60 leukaemia cells was included as a positive control. Lane 1 - untreated HL60 cells, Lane 2- etoposide treated HL60 cells, Lane 3 - CXR9 treated SK-OV3 cells, Lane 4 - HXR9 treated SK-OV3 cells. 'PARP' - position of full length PARP protein (116kDa), 'PARP(c)' - position of cleaved PARP (85kDa). Beta-actin is included as a loading control.

Further, immunoblotting indicated that there was a reduction in full-length PARP in HXR9-treated SK-OV3 cells (Fig 5); PARP cleavage is another characteristic event of apoptosis [14].

**Figure 6:**
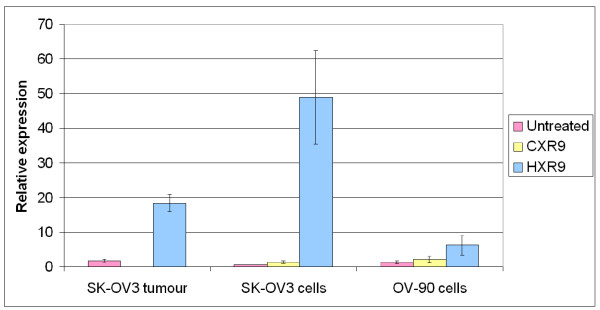
**c-Fos expression is elevated after treatment with HXR9**. SK-OV3 and OV-90 cells were incubated for 24 hours with 120 μM HXR9 or CXR9 and *cFos *expression was analysed by RT-QPCR. RNA was also extracted from SK-OV3 tumours excised from mice treated twice weekly with HXR9 (10 mg/Kg)*iv*, after an initial dose of 100 mg/Kg *iv *on day 3. Results are presented as a ratio with *beta-actin *expression (x1000). A very high increase in transcription of *c-Fos *in HXR9 treated SK-OV3 cells and tumours was observed. Error bars represent the standard error of the mean for three independent experiments (cell lines) or for three tumours (*in vivo *model).

Previous studies have shown that upregulation of *cFos *is a key event in HXR9-mediated apoptosis in both melanoma and renal cancer cells [6,7]. Semi-quantitative PCR revealed that *cFos *expression is on average 50 times greater in SK-OV3 cells treated with HXR9 (Fig 6) than in either untreated or CXR9-treated cells, whilst HXR9 treatment of OV-90 cells did not cause a significant increase in cFos expression (Fig 6).

### HXR9 retards tumour growth *in vivo*

**Figure 7:**
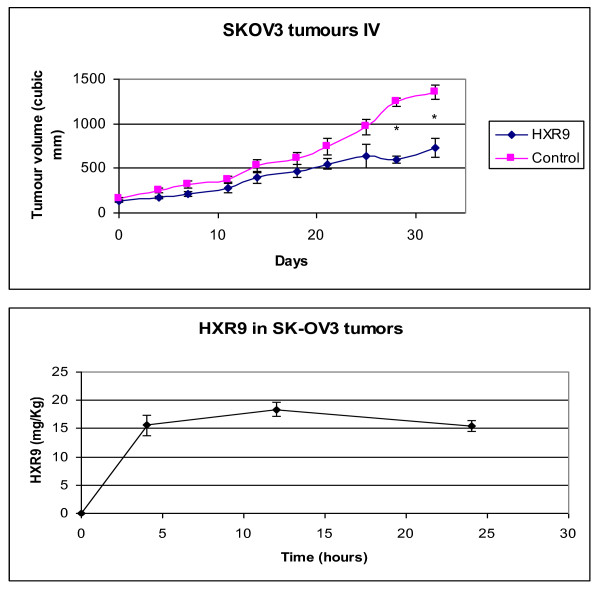
**(a) HXR9 retards SK-OV3 tumour growth in mice**. Mice were treated twice weekly with HXR9 (10 mg/Kg)*iv*, after an initial dose of 100 mg/Kg *iv *when the mean tumour volume reached 100 mm^3^. Error bars represent the SEM for each data point. * indicates a significant difference between treatment groups, p < 0.05 (Mann-Whitney test). **(b) ***HXR9 accumulation in SK-OV3 tumours*. Tumours were removed from mice at 4 hours, 12 hours and 24 hours after an initial dose of HXR9 at 100 mg/Kg *iv *and the amount of HXR9 in the tumour tissue was assessed using dot blotting with an anti-HXR9 antibody.

In order to assess the efficacy of HXR9 *in vivo *we established a xenograft model of SK-OV3 in nude mice by injecting cells into the flank. When the average tumour volume had reached 100 mm^3 ^mice were given an initial dose of HXR9 of 100 mg/Kg *iv*, followed by twice weekly *iv *treatments at 10 mg/Kg. Tumour growth in HXR9 treated mice was retarded by 46% at 32 days compared to the control group (Fig 7a). RNA was extracted from tumours at the end of 32 days in order to measure *cFos *expression by QPCR; *cFos *was found to be expressed at an 18-fold higher level in tumours from HXR9 treated mice than in tumours from untreated mice (Fig 6).

The uptake of HXR9 by tumours after the initial dose was measured by excising tumours at 4, 12 and 24 hours and assessing the amount of HXR9 using an anti-HXR9 antibody. This indicated that there is a rapid accumulation of peptide in the first four hours and that the amount of peptide remains stable beyond 24 hours (Fig 7b).

## Discussion

It is becoming increasingly apparent that *HOX *genes are frequently dysregulated in different malignancies [15-17] including renal [7], bladder [18], prostate [19,20], lung [21] and ovarian cancer [9-11] (this study). The exact pattern of *HOX *expression varies for different malignancies and the significance of this is worthy of further investigation. Possible explanations may lie in the original expression of *HOX *genes in the embryonic cells, in which case the *HOX *expression profile would influence the phenotype of each cancer. There is clear evidence for this in ovarian cancer where the expression of *HOXA9*, *HOXA10 *or *HOXA11 *confer a serous, endometrioid-like and mucinous-like phenotype respectively [9]. It is also clear that the over-expression of some HOX genes is directly related to the degree of malignancy, for example elevated expression of *HOXC8 *is correlated with the loss of differentiation of prostate tumours [20], although our data indicate that *HOXC8 *is actually expressed at a lower level in the ovarian cancer lines SK-OV3 and OV-90 as compared to normal ovarian tissue. In addition, loss of *HOXA5 *and *HOXA9 *expression is generally associated with an increased resistance to apoptosis and tumour survival [22], although both of these genes are expressed at relatively high levels in SK-OV3 compared to normal ovarian tissue and OV-90. However, recent studies have shown that *HOXA9 *expression increases considerably in advanced stages of ovarian cancer, indicating that it may have a different function as the disease progresses [23]. The other *HOX *genes that are notably upregulated in SK-OV3 cells (but generally not OV-90 cells) have previously been shown to be upregulated in other cancers, including *HOXA13 *[24], *HOXB5 *[23], *HOXB9 *[25] and *HOXD*9 [26].

One difficulty in interpreting this data is that, in general, there is a high level of redundancy in *HOX *function which may mask the contribution of any of these individual genes to a malignant phenotype. Hence a post-translational approach may reveal more about global *HOX *functions in cancer. The HXR9 peptide blocks the binding of HOX proteins to their PBX co-factor and in doing so alters the way in which they bind to DNA and hence changes the regulation of their target genes [6,7]. HXR9 causes melanoma and renal cancer cells to undergo apoptosis, and here we have shown that the SK-OV3 cell line derived from an ovarian tumour responds in the same way, indicating that the HOX/PBX interaction is a potential target in ovarian cancer therapy. Interestingly, HXR9 does not induce cell death in OV-90 cells that generally do not show dysregulated HOX expression.

The role of *HOX *genes in early development is highly conserved [2]. They may also have a common role in malignant cells, as evidenced by a common sensitivity to HXR9 and in similar transcriptional responses, including the upregulation of *cFos*. This change in *cFos *expression was previously shown to be responsible, at least in part, for the induction of apoptosis [6]. Recent studies have shown that *cFos *transcriptionally represses the key anti-apoptotic gene *c-FLIP(L)*, greatly sensitising prostate cancer cells to TRAIL-induced apoptosis [27,28].

## Conclusions

Novel targets are needed in ovarian cancer therapy as the long term survival of patients is poor despite the trial of numerous chemotherapeutic regimes including the current standard of care, carboplatin/paclitaxel [1,29]. The *HOX *genes are independent of the targets of current chemotherapeutics (as far as this can be known), and hence targeting the HOX/PBX interaction is a potential alternative or addition to current therapeutics when drug resistance develops.

## Competing interests

The authors declare that they have no competing interests.

## Authors' contributions

All authors have read and approved the final manuscript. The authors made the following contributions to this work: LP conducted lab based experimental work, KJH was involved in experimental design and critique, AM was in charge of sample acquisition and critique, HSP helped to write the manuscript and RM was involved in experimental design and also wrote the manuscript. All authors read and approved the final paper.

## Pre-publication history

The pre-publication history for this paper can be accessed here:

http://www.biomedcentral.com/1471-2407/10/89/prepub
